# Brief compression-only cardiopulmonary resuscitation training video and simulation with homemade mannequin improves CPR skills

**DOI:** 10.1186/s12873-016-0110-5

**Published:** 2016-11-29

**Authors:** Gregory K. Wanner, Arayel Osborne, Charlotte H. Greene

**Affiliations:** 1 Clinical Faculty, Department of Emergency Medicine, Christiana Care Health System, 4755 Ogletown-Stanton Road, Newark, DE 19718 USA; 2 Philadelphia College of Osteopathic Medicine, Philadelphia, PA 19131 USA; 3 Department of Biomedical Sciences, Philadelphia College of Osteopathic Medicine, 4170 City Avenue, Philadelphia, PA 19131 USA

## Abstract

**Background:**

Cardiopulmonary resuscitation (CPR) training has traditionally involved classroom-based courses or, more recently, home-based video self-instruction. These methods typically require preparation and purchase fee; which can dissuade many potential bystanders from receiving training. This study aimed to evaluate the effectiveness of teaching compression-only CPR to previously untrained individuals using our 6-min online CPR training video and skills practice on a homemade mannequin, reproduced by viewers with commonly available items (towel, toilet paper roll, t-shirt).

**Methods:**

Participants viewed the training video and practiced with the homemade mannequin. This was a parallel-design study with pre and post training evaluations of CPR skills (compression rate, depth, hand position, release), and hands-off time (time without compressions). CPR skills were evaluated using a sensor-equipped mannequin and two blinded CPR experts observed testing of participants.

**Results:**

Twenty-four participants were included: 12 never-trained and 12 currently certified in CPR. Comparing pre and post training, the never-trained group had improvements in average compression rate per minute (64.3 to 103.9, *p* = 0.006), compressions with correct hand position in 1 min (8.3 to 54.3, *p* = 0.002), and correct compression release in 1 min (21.2 to 76.3, *p* < 0.001). The CPR-certified group had adequate pre and post-test compression rates (>100/min), but an improved number of compressions with correct release (53.5 to 94.7, *p* < 0.001). Both groups had significantly reduced hands-off time after training. Achieving adequate compression depths (>50 mm) remained problematic in both groups. Comparisons made between groups indicated significant improvements in compression depth, hand position, and hands-off time in never-trained compared to CPR-certified participants. Inter-rater agreement values were also calculated between the CPR experts and sensor-equipped mannequin.

**Conclusions:**

A brief internet-based video coupled with skill practice on a homemade mannequin improved compression-only CPR skills, especially in the previously untrained participants. This training method allows for widespread compression-only CPR training with a tactile learning component, without fees or advance preparation.

## Background

Each year over 300,000 people in the United States experience out-of-hospital cardiac arrest [1]. Cardiopulmonary resuscitation (CPR) initiated by bystanders can significantly improve survival outcomes, however, only 46.1% of cardiac arrest victims initially receive CPR from a bystander [1–3]. To help simplify the steps of CPR, compression-only CPR was included in the 2010 American Heart Association (AHA) guidelines as an effective method for untrained bystanders [4–8].

CPR training has traditionally consisted of an instructor-led course lasting several hours and requiring a course fee. Unfortunately the planning, time commitment, and cost all have the potential to dissuade many people from receiving CPR training [9, 10]. More recently, abbreviated training methods providing video instruction and practice on an inflatable mannequin have proven to be effective [11–15]. While instructor-led training is recommended, European guidelines suggest that self-instruction with hands-on practice appears to be an “effective alternative” [11].

Our goal was to evaluate the effectiveness of using an internet-based video along with a homemade practice mannequin, reproduced by participants, to teach the skills of compression-only CPR to previously untrained individuals.

## Methods

### Study design

This was a parallel-design study with pre and post training evaluations of CPR effectiveness.

### Participant recruitment and exclusion

Employees, students, and visitors at the Philadelphia College of Osteopathic Medicine were recruited via electronic billboards, campus-wide emails, and word of mouth. Individuals either never previously formally trained in CPR (“untrained”) or currently certified in CPR (“trained/certified”) were eligible for inclusion. This study aimed to evaluate a brief training session on previously untrained individuals’ CPR skills. The CPR certified group was included as a comparison group to evaluate the effects of our training, if any, on individuals judged to be competent in CPR, in relation to untrained individuals. Previously CPR-trained but not currently CPR certified individuals were excluded. Demographics of participants are included in Table 1.

**Table 1:** Demographics and CPR experience of participants

Characteristic	Untrained (*N* = 12)	Trained/Certified (*N* = 12)
Age, mean years (SD)	28.1 (12.3)	25.8 (4.0)
Male gender (%)	25.0	58.3
Status (%)		
Medical student	0	75.0
Graduate student	25.0	8.3
Employee (non-clinical)	33.3	0
Visitor	41.7	16.7
Total time CPR certified (years)	–	2.3
Last CPR course (months ago)	–	5.1
Witnessed CPR, ever, %	16.7	33.3
Performed CPR, ever, %	0	16.7

### Intervention

**Fig. 1:**
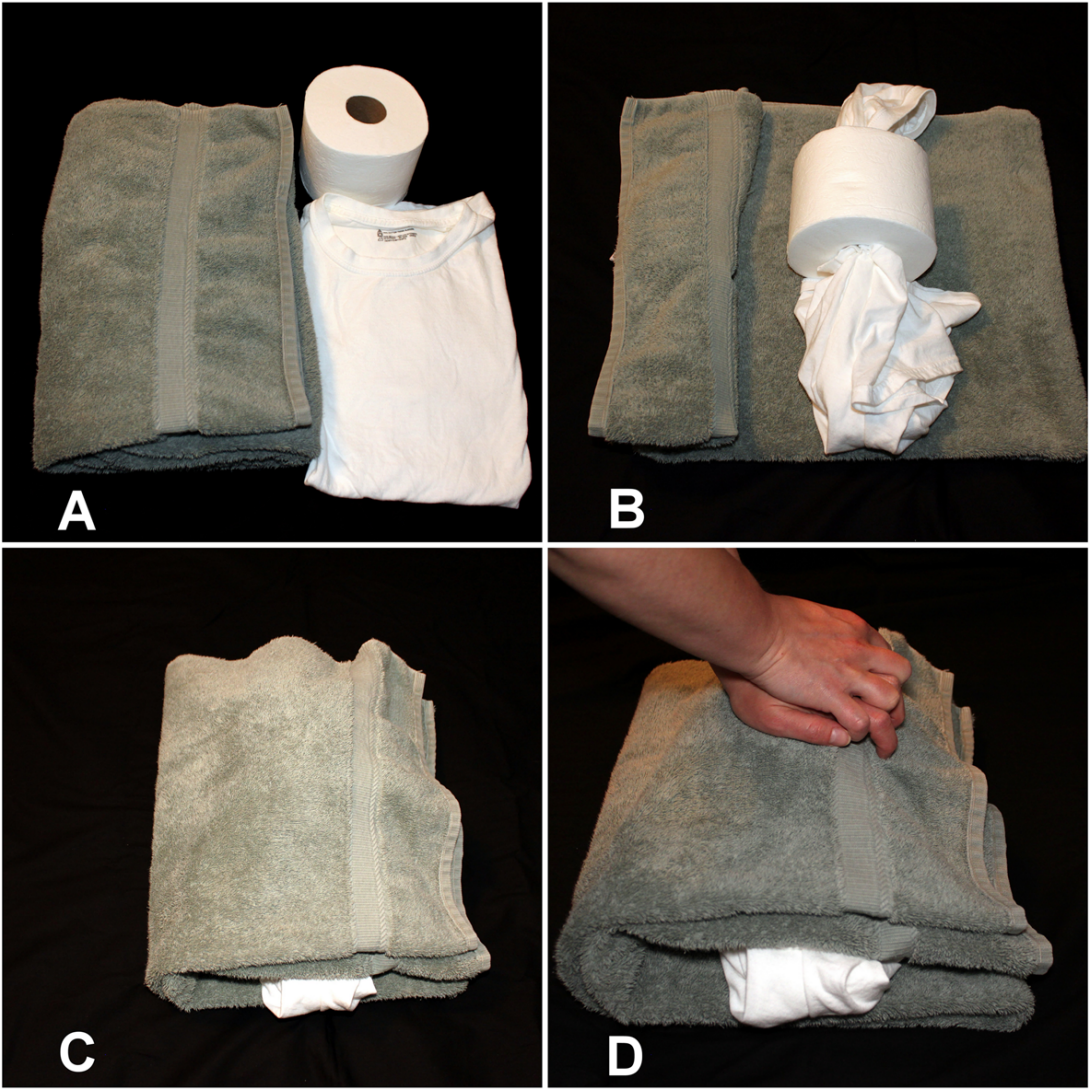
**a** Materials to build CPR tool (towel, toilet paper, T-shirt); **b** T-shirt is placed within toilet paper tube; **c** Toilet paper roll/shirt are folded into towel; **d** Practicing compression-only CPR

All participants viewed a 6-min CPR training video we produced consisting of basic information about compression-only CPR, a CPR demonstration, instruction on producing a homemade CPR mannequin (CPR tool), and encouragement to practice along with the video [16]. The CPR tool was recreated by participants using common household items (towel, toilet paper roll, and t-shirt), provided to participants (Fig. 1). Our goal was to evaluate whether participants would be able to recreate and use a homemade CPR tool along with video instruction to improve CPR skills, rather than to extensively evaluate the CPR tool itself. Materials for the homemade CPR tool were chosen after subjectively evaluating several other commonly available items, including pillows and plastic bottles. We compared our homemade CPR tool to the CPR Anytime kit (AHA, Dallas, TX), which required approximately 35.5 kg of force to cause a “click” signaling adequate depth. With this compressive force on our homemade mannequin, an average of 46.6 mm of depth was attained during compressions.

### Outcome measures

All participants were given an identical scenario in which “a man collapses in the classroom.” Participants were allowed 1-min to “perform as if this were a real situation” and evaluated using a sensor-equipped CPR mannequin (ResusciAnne® SkillReporter™) before and after the training video. Additionally, two blinded CPR-experts (emergency physicians, one current and one former CPR instructor) viewed videos of participants performing CPR. The video viewing order was randomized, blinding the CPR experts to the group (untrained versus CPR certified) and also blinding to the pre or post-training status of study participants. The CPR experts observed for appropriately performed skills (compression rate, depth, and recoil), similarly to a traditional CPR class; however, the sensor-equipped mannequin’s objective data was used for data analysis. Primary outcome measures included compression rate, compression depth, correct hand position, correct release, and total hands-off time during the 1-min testing period. Secondary outcome measures included participants’ opinions about their willingness to perform compressions. Inter-rater reliability was also evaluated between the CPR experts and sensor-equipped mannequin.

### Statistical methods

Sample size estimates were calculated using a power analysis to predict that a statistically significant difference could be detected at the alpha = 0.05 level with a power of 80%. Paired-difference t-test was used to evaluate the difference in performance of each group before and after training. Comparisons were made between groups using one-factor analysis of variance (ANOVA). Inter-rater reliability (Fleiss kappa) calculations were used to evaluate the CPR experts’ and sensor-equipped mannequin’s data [17]. Statistical analysis was performed using SAS software (SAS, Cary, NC).

## Results

Comparing pre and post training, both the untrained group and trained/certified group showed improvements in several skills, reported in Table 2. In the untrained group, significant improvements were seen in compression rate, correct hand position, correct compression release/recoil, and hands off time. The trained/certified group had adequate compression rates (>100/min) before and after training. Comparing pre and post training, the trained/certified group had significant improvements in compressions with correct release, hands off time, and time to first compression. Compression depth remained inadequate (<50 mm) in both groups.

**Table 2:** Results before and after training in untrained and trained/certified groups

	Pre-training	Post-training	*p*-value
Untrained (*n* = 12)			
Compression rate per minute (SD)	64.3 (43.6)	103.9 (20.7)	0.006
Compression depth, mean in mm (SD)	26.8 (17.1)	35.4 (11.2)	0.19
Correct hand position, compression # in 1 min (SD)	8.3 (6.3)	54.3 (41.4)	0.002
Correct release, in 1 min (SD)	21.2 (20.4)	76.3 (16.3)	<0.001
Hands-off time, seconds in 1 min (SD)	41.8 (14.6)	15.3 (8.3)	<0.001
Time to first compression, seconds (SD)	18.0 (15.1)	15.3 (8.3)	0.60
Trained/Certified (*n* = 12)			
Compression rate per minute (SD)	119.3 (15.2)	120.7 (12.8)	0.65
Compression depth, mean in mm (SD)	46.3 (10.4)	43.8 (9.8)	0.02
Correct hand position, compression # in 1 min (SD)	41.5 (25.7)	59.3 (49.3)	0.15
Correct release, in 1 min (SD)	53.5 (19.3)	94.7 (15.4)	<0.001
Hands-off time, seconds in 1 min (SD)	28.8 (7.1)	11.4 (5.3)	<0.001
Time to first compression, seconds (SD)	17.4 (7.8)	11.1 (2.5)	0.02

### Group comparisons

ANOVA testing revealed several significant findings, reported in Table 3. The compression rate per minute in the untrained group was significantly different both before and after training, compared to the trained/certified group. Although a statistically significant difference remained after training, both groups performed at adequate compression rates (>100 per minute, per AHA guidelines). The number of compressions with correct hand position in 1 min was significantly different prior to training comparing untrained and trained/certified groups; however, after training the difference was no longer significant. Similarly, regarding both compression depth and hands-off time in 1 min, prior to training there were significant differences between untrained and trained/certified groups; however, post-training comparisons of groups revealed a non-significant difference in compression depth and hands-off time.

**Table 3:** Comparisons between groups, before and after training

	Pre/post training	Untrained	Trained/certified	*p*-value*
Compression rate, per minute (SD)	Pre	64.3 (43.6)	119.3 (15.2)	<0.001
	Post	103.9 (20.7)	120.7 (12.8)	0.026
Compression depth, mm (SD)	Pre	26.8 (17.1)	46.3 (10.4)	0.003
	Post	35.4 (11.2)	43.8 (9.8)	0.062
Correct hand position, per minute (SD)	Pre	8.3 (6.3)	41.5 (25.7)	<0.001
	Post	54.3 (41.4)	59.3 (49.3)	0.790
Hands-off time, seconds in 1 min (SD)	Pre	41.8 (14.6)	28.8 (7.1)	0.011
	Post	15.3 (8.3)	11.4 (5.3)	0.193

* Results of analysis of variance (ANOVA) testing

### Evaluators’ ratings

Two blinded CPR experts viewed videos of each participant, observing participants’ CPR performance for correct compression rate, depth, and full chest recoil; based on the AHA 2010 CPR guidelines. These items were also reported by the CPR mannequin’s sensors. Inter-rater agreement was judged by calculating a Fleiss kappa value (κ). Overall inter-rater agreement between the two human graders (CPR experts) for correctly performed skills was 65.9% (κ = 0.298). Overall inter-rater agreement between the CPR experts and the mannequin’s computer was fair (κ = 0.335).

### Participants’ opinions

Surveys were completed by participants before and after training, including two questions to assess their opinions of performing CPR. Questions were answered using a five-point Likert scale (1 = Strongly Disagree, 5 = Strongly Agree). Responding to the question “How comfortable do you feel in your ability to perform chest compressions?” both groups reported significant improvements in their self-perceived comfort levels. Answering the question “If you saw someone suddenly collapse, would you perform chest compressions, if needed?” showed non-significant increases in willingness to perform compressions. Results are reported in Table 4.

**Table 4:** Responses to survey questions

Survey question (5 = Strongly Agree, 1 = Strongly Disagree)			
	Pre-training	Post-training	*p*-value
“How comfortable do you feel in your ability to perform chest compressions?”			
Untrained	1.33	2.83	0.010
Trained/Certified	2.67	3.75	0.008
“If you saw someone suddenly collapse, would you perform chest compressions, if needed?”			
Untrained	2.42	3.08	0.07
Trained/Certified	3.75	3.83	0.59

## Discussion

Prior studies have shown the effectiveness of video-based brief CPR training compared to traditional CPR courses [12, 18, 19]. Our 6-min training video, production of homemade mannequin, and practice with this tool resulted in improvement in several aspects of CPR performance in both the untrained group and trained/certified group. We noted improvements in compression rate, hand position, recoil, and hands-off time—especially in untrained participants—but difficulties with adequate depth in both groups. These results are similar to prior studies of brief CPR training, including brief classroom courses and video-based training lasting between 60 s and 22 min [13, 14, 20]. Our study appears to be the first to include a homemade CPR mannequin with a self-directed instructional video; successfully adding a tactile component to home-based training. Additionally, participants’ self-perceived abilities improved after training, but their willingness to perform compressions did not significantly change.

### Limitations

Demographic differences were present between the two testing groups, including notable differences in gender, status (student, employee, visitor), and age ranges. It is unclear if these differences may have influenced our results. We did not evaluate CPR skill retention, however prior studies have suggested adequate retention after brief training [13, 19]. Participants performed identical testing scenarios before and after training, increasing familiarity with the testing mannequin, and potentially influencing results. The trained/certified group was included to help control for this effect, as this group has had prior experience with CPR and mannequin use, allowing a better understanding of the true effect of training on previously untrained participants. The inter-rater agreement between the two CPR experts was fairly low overall, highlighting the possible limitations of human graders’ evaluations. To reduce potential for subjectivity, the objective data obtained from the sensor-equipped CPR mannequin was used for statistical analysis; as was planned in the study design. The homemade mannequin/CPR tool was observed to be easily recreated by participants, however we did not formally evaluate participants’ opinions of the CPR tool. Researchers observed that the toilet paper roll began to fatigue after several compressions, producing less recoil. Further research could focus on improving recoil in an equally convenient homemade CPR tool, improving compression depth, and directly comparing the homemade CPR tool to a commercially available CPR mannequin.

## Conclusions

We feel it is possible to teach the basics of compression-only CPR by combining a brief video and simulation with a homemade mannequin. Our method used a convenient internet-based video and a mannequin reproduced by viewers, requiring only an internet-enabled device and a towel, roll of toilet paper, and a t-shirt. While previously untrained participants appeared to benefit most from our training—performing similarly to CPR-certified participants in terms of hand position, depth, and hands-off time—the trained/certified participants also improved some skills; suggesting a possible role as a home-based refresher. Adequate compression depth remained problematic for both groups. These results suggest that using a brief online CPR video with a homemade mannequin has the ability to teach basic compression-only CPR skills with no additional cost or advance preparation.
